# Age-Dependent Contributions of NMDA Receptors and L-Type Calcium Channels to Long-Term Depression in the Piriform Cortex

**DOI:** 10.3390/ijms222413551

**Published:** 2021-12-17

**Authors:** Vishaal Rajani, Aida Maziar, Kwun Nok Mimi Man, Johannes W. Hell, Qi Yuan

**Affiliations:** 1 Biomedical Sciences, Faculty of Medicine, Memorial University of Newfoundland, St. John’s, NL A1B 3V6, Canada; amaziar@mun.ca; 2 Department of Pharmacology, School of Medicine, University of California at Davis, Davis, CA 95616-5270, USA; knmman@ucdavis.edu (K.N.M.M.); jwhell@ucdavis.edu (J.W.H.)

**Keywords:** NMDA receptor, L-type calcium channel, long-term depression, piriform cortex, rat

## Abstract

In the hippocampus, the contributions of N-methyl-D-aspartate receptors (NMDARs) and L-type calcium channels (LTCCs) to neuronal transmission and synaptic plasticity change with aging, underlying calcium dysregulation and cognitive dysfunction. However, the relative contributions of NMDARs and LTCCs in other learning encoding structures during aging are not known. The piriform cortex (PC) plays a significant role in odor associative memories, and like the hippocampus, exhibits forms of long-term synaptic plasticity. Here, we investigated the expression and contribution of NMDARs and LTCCs in long-term depression (LTD) of the PC associational fiber pathway in three cohorts of Sprague Dawley rats: neonatal (1–2 weeks), young adult (2–3 months) and aged (20–25 months). Using a combination of slice electrophysiology, Western blotting, fluorescent immunohistochemistry and confocal imaging, we observed a shift from an NMDAR to LTCC mediation of LTD in aged rats, despite no difference in the amount of LTD expression. These changes in plasticity are related to age-dependent differential receptor expression in the PC. LTCC Cav1.2 expression relative to postsynaptic density protein 95 is increased in the associational pathway of the aged PC layer Ib. Enhanced LTCC contribution in synaptic depression in the PC may contribute to altered olfactory function and learning with aging.

## 1. Introduction

The piriform cortex (PC) is a central component of olfactory information processing, underlying odor discrimination and contextualization [1,2]. Situated in the ventrolateral forebrain, the PC has a laminar structure, consisting of layers of pyramidal and semilunar neurons (layers II and III) whose dendrites extend out to the lateral olfactory tract (LOT) near the tissue surface, forming synaptic connections in adjacent layers. The inner synaptic layer (Ib) consists of associational cortico-cortical synapses, while the outer layer (Ia) makes synaptic connections with the LOT, passing afferent olfactory information via fibers from the olfactory bulb [3]. In conjunction with its role in olfactory encoding, the PC is a site involved in associative memory formation, exhibiting a high degree of synaptic plasticity during early developmental periods, which moderately persists into adulthood [4,5,6,7].

Critical to synaptic plasticity within the piriform cortex are N-methyl-D-aspartate receptors (NMDARs) and voltage-gated L-type calcium channels (LTCCs), which initiate diverse calcium-dependent signalling cascades that underlie the molecular basis of associative memory across different developmental stages. Sharing homology with the hippocampus, high- and low-frequency stimulation of afferent and associational fibers within the PC produce patterned calcium influx via NMDARs to induce long-term potentiation (LTP) or long-term depression (LTD) of synaptic activity, respectively [4,7,8,9,10]. In the PC afferent sensory pathway, NMDARs undergo a rapid activity-dependent postnatal down-regulation [11], corresponding to a brief window where NMDAR-dependent LTP can be induced reliably [7]. However, the associational pathway remains highly plastic into adulthood [7,9], consistent with its primary role in associative memory [2].

The role of LTCCs in PC plasticity such as LTP and LTD has not been studied exclusively. However, PC LTCCs are critical for early odor preference learning in rodents, a form of associative learning only occurring within an early postnatal period [6,12]. The differential roles of NMDARs and LTCCs are studied in this model. While NMDARs are critical in initiating odor-specific memory encoding, LTCCs mediate long-term memory storage. Blocking PC LTCCs during associative learning specifically impairs long-term memory while leaving short-term memory intact [6]. However, how the roles of NMDARs and LTCCs evolve during development and into aging are not known.

In the hippocampus, LTCC expression and function increase with aging. In aging rabbit hippocampal cornu Ammonis 1 (CA1) neurons, an increase in LTCC function produces a larger afterhyperpolarization and increased calcium action potentials which are suggested to contribute to reduced neuronal excitability and decreased learning performance [13,14,15,16,17]. Moreover, the age-related increase in LTCCs is associated with a shift in forms of synaptic plasticity in aged rats, exhibiting a reduced NMDAR-dependent and increased LTCC-dependent LTP and LTD at Schaffer-collateral-CA1 synapses when compared to young rats [18,19,20]. This age-related modification of presence and function of LTCCs in hippocampal neurons could contribute to dysregulated calcium homeostasis, resulting in synaptic dysfunction and cognitive decline [21], although a protective role of increased LTCC plasticity in aging has also been proposed [18,19].

Here, we investigated the age-dependent roles of NMDARs and LTCCs in synaptic plasticity of the anterior PC. By assessing LTD within the associational layer Ib of neonatal, adult and aging rats, we report that, similar to the hippocampus, the PC exhibits an age-dependent shift in relative receptor contribution and downstream signalling cascades to synaptic plasticity.

## 2. Results

### 2.1. Age-Dependent Shifts in NMDAR and LTCC Contribution to LTD in Layer Ib of the Piriform Cortex

**Figure 1:**
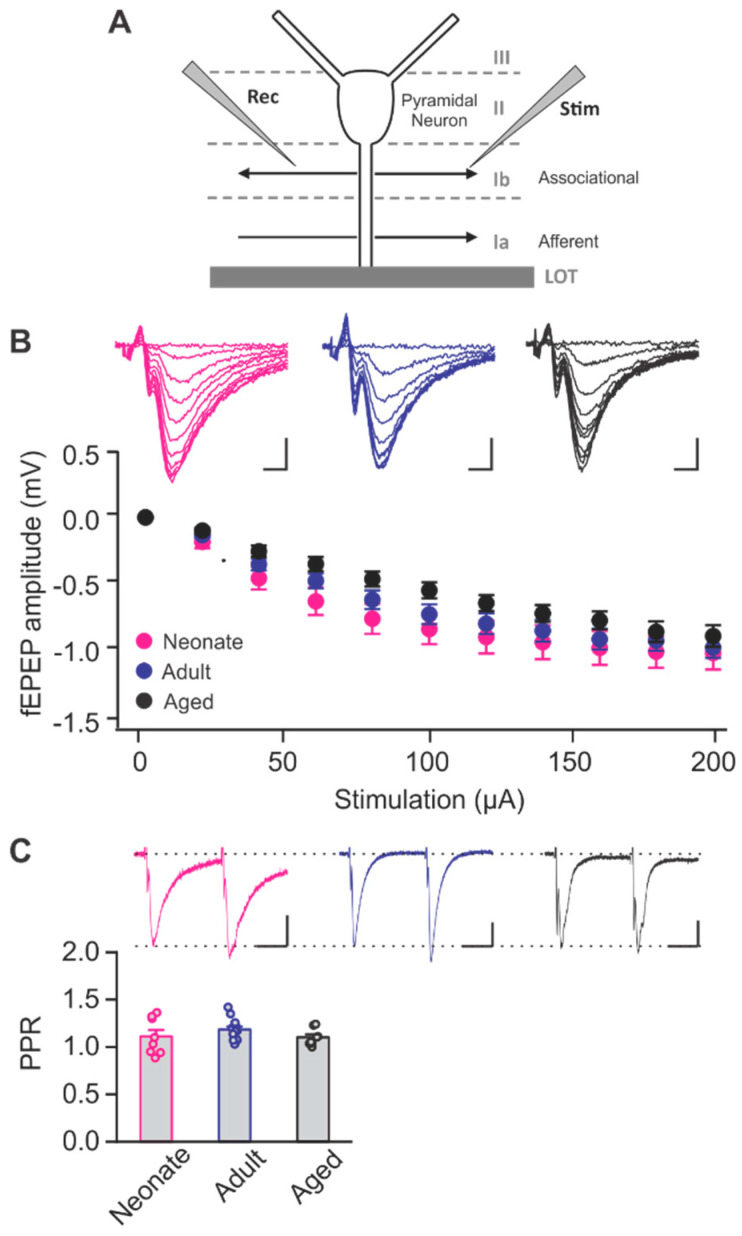
Input/output relationship and release properties of piriform layer Ib at different ages. (**A**) Schematic of the piriform cortex layers and recording configuration. (**B**) Input (stimulation intensity) vs. output (fEPSP peak amplitude) relationship in layer Ib. Example traces from different ages are shown in the upper panel. Neonate: *n* = 15; Adult: *n* = 12, Aged: *n* = 16. Scale bars, 0.5 mV, 2 msec. (**C**) Paired pulse ratios (PPRs) of fEPSPs at different ages. Neonate: *n* = 8; Adult: *n* = 13, Aged: *n* = 8. Scale bars, 0.2 mV, 20 msec. Stimulation artifacts were truncated in all example traces.

To assess the effect of aging on the roles of NMDARs and LTCCs in synaptic plasticity of the PC, we investigated field excitatory post synaptic potentials (fEPSPs) in PC slices from three different age cohorts: neonatal (1–2 weeks), adult (2–3 months) and aged (20–25 months). Synaptic responses were evoked by stimulating associational fibers in layer Ib of the PC (Figure 1A). The fEPSP evoked exclusively by associational fiber stimulation was verified by its sensitivity to γ-aminobutyric acid (GABA)_B_ receptor agonist baclofen [11]. Baclofen greatly reduced fEPSP and increased paired pulse ratio (PPR) evoked by Ib, but not LOT stimulation (Supplementary Figure S1). Examination of input/output relationships based on stimulation intensity showed no significant difference in fEPSP amplitudes between age groups (F_2,40_ = 2.046, *p* = 0.160, two-way repeated analysis of variance (ANOVA), Figure 1B). To determine whether age had any impact on presynaptic release properties, we tested the PPR of fEPSPs, and found PPRs were not significantly different across the three age cohorts (F_2,26_ = 1.238, *p* = 0.306, one-way ANOVA, Figure 1C).

**Figure 2:**
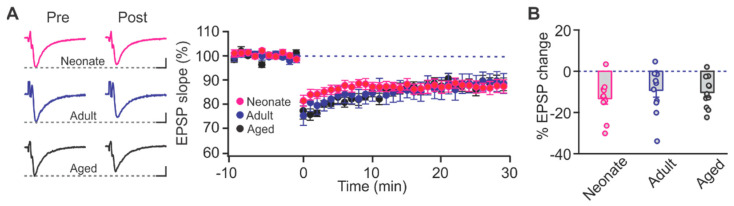
Long-term depression (LTD) in the piriform cortex layer Ib. (**A**) Example traces and time courses of fEPSPs with LTD inductions in neonate, adult and aged animals. *n* = 11 for each age group. Scale bars, 0.5 mV, 5 msec. (**B**) Percentage changes of fEPSPs at 30 min post-induction.

To induce LTD, we implemented a low-frequency stimulation (LFS) protocol, 1 Hz for 15 min (900 stimuli), which produced significant and sustained decreases in synaptic activity 30 min post-induction compared to the baseline in all age groups: (neonatal: t = 4.086, *p* = 0.002, adult: t = 2.863, *p* = 0.017, and aged: t = 5.018, *p* < 0.001; paired *t*-test; Figure 2A). However, there was no significant difference in the amount of LTD expressed at different ages (F_2,30_ = 0.552, *p* = 0.582; One-way ANOVA; Figure 2B).

**Figure 3:**
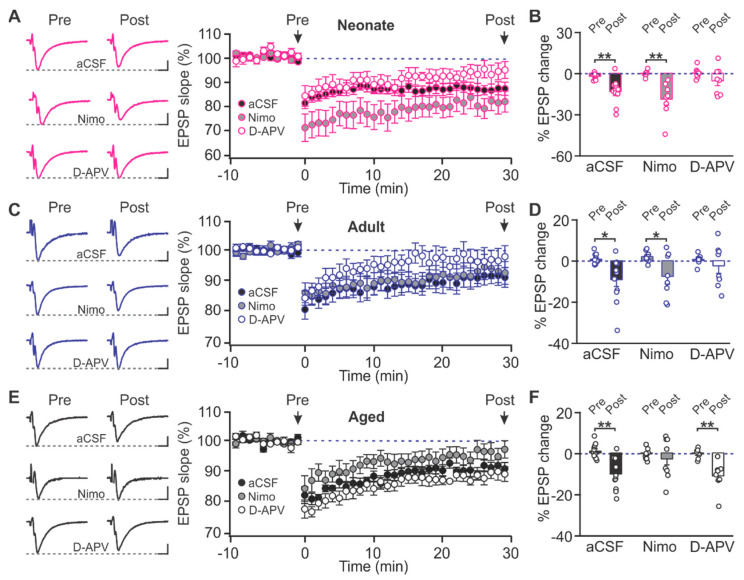
Age-dependent LTD compositions in the piriform cortex layer Ib. (**A**,**B**) Example traces and time courses of fEPSPs with LTD inductions in aCSF, nimodipine (Nimo) and D-APV (**A**), and % changes of fEPSPs at 30 min post-LTD induction (**B**) in neonate rats. aCSF: *n* = 11; nimo: *n* = 8, APV: *n* = 8. (**C**,**D**) Time courses with LTD inductions and % changes of fEPSPs in adult rats. aCSF: *n* = 11; nimo: *n* = 8, APV: *n* = 8. (**E**,**F**) Time courses with LTD inductions and % changes of fEPSPs in aged rats. aCSF: *n* = 11; nimo: *n* = 10, APV: *n* = 8. Scale bars, 0.5 mV, 5 msec. * *p* < 0.05; ** *p* < 0.01.

We then looked at the relative contributions of the NMDARs and LTCCs in LTD induction at different ages. In the neonate, LTD was intact in the presence of the LTCC antagonist nimodipine (10 µM) (t = 4.547, *p* = 0.003, pre vs. post), but was prevented by NMDAR blockade by D-APV (50 µM) (t = 1.865, *p* = 0.104, pre vs. post; Figure 3A,B). Similarly, in the adult rats, D-APV (t = 0.877, *p* = 0.410, pre vs. post), but not nimodipine (t = 2.695, *p* = 0.031, pre vs. post), prevented LTD induction (Figure 3C,D). Intriguingly, in the aged rats, nimodipine (t = 0.948, *p* = 0.368, pre vs. post), but not D-APV (t = 3.507, *p* = 0.0099, pre vs. post), blocked LTD induction (Figure 3E,F). These results suggest that with aging, there is a reduced contribution of NMDAR-dependent LTD within the associational fiber layer in the PC, which is replaced by a dependency on LTCC-mediated LTD.

### 2.2. The Effects of Aging on NMDAR and LTCC Expression in the Piriform Cortex

**Figure 4:**
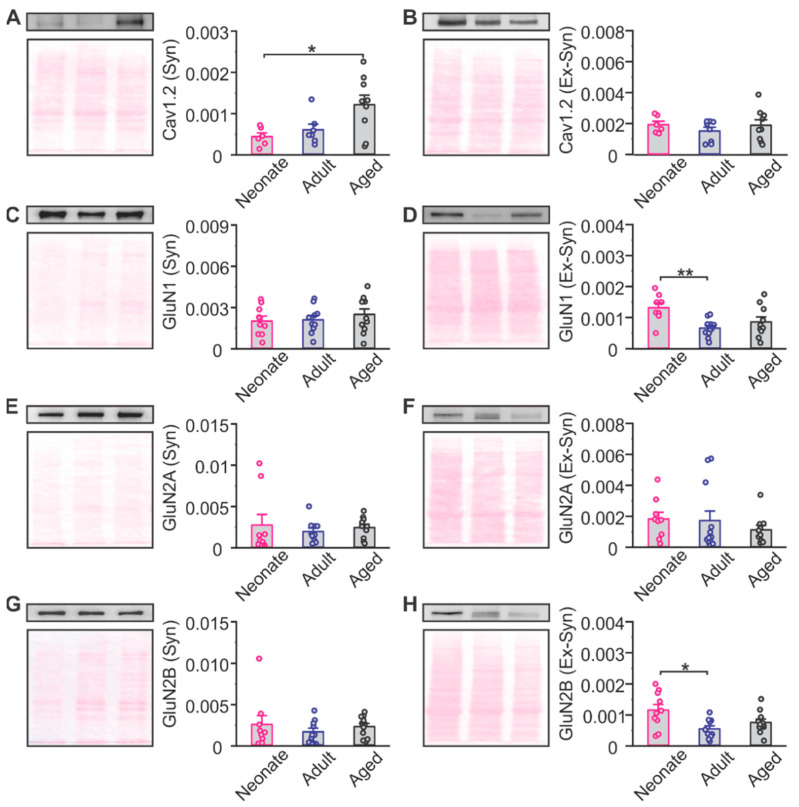
Age-dependent calcium permeable channel expressions in the piriform cortex. (**A**) Synaptic expressions of L-type calcium channel Cav1.2 subunit. Neonate: *n* = 6; Adult: *n* = 7, Aged: *n* = 9. (**B**) Extra-synaptic expressions of Cav1.2 subunit. Neonate: *n* = 6; Adult: *n* = 8, Aged: *n* = 9. (**C**) Synaptic expressions of NMDAR GluN1 subunit. Neonate: *n* = 9; Adult: *n* = 10, Aged: *n* = 11. (**D**) Extra-synaptic expressions of GluN1 subunit. Neonate: *n* = 8; Adult: *n* = 11, Aged: *n* = 10. (**E**) Synaptic expressions of NMDAR GluN2A subunit. Neonate: *n* = 9; Adult: *n* = 10, Aged: *n* = 10. (**F**) Extra-synaptic expressions of GluN2A subunit. Neonate: *n* = 9; Adult: *n* = 12, Aged: *n* = 10. (**G**) Synaptic expressions of NMDAR GluN2B subunit. Neonate: *n* = 9; Adult: *n* = 10, Aged: *n* = 11. (**H**) Extra-synaptic expressions of GluN2B subunit. Neonate: *n* = 10; Adult: *n* = 10, Aged: *n* = 11. * *p* < 0.05; ** *p* < 0.01.

In the hippocampus, there is an age-related decrease in NMDAR expression concurrent with an increase in LTCCs, which is reflected in the changes in receptor contribution to synaptic plasticity [18,19,20]. To determine the age-related expression of NMDARs and LTCCs in the PC, we first examined synaptic and extra-synaptic expression of the Cav1.2 subunit of the LTCC, and the GluN1, GluN2A and GluN2B subunits of the NMDAR of the PC extracts using Western blotting (Figure 4). We found that there was a significant increase in synaptic Cav1.2 in aged rats when compared to neonates (F_2,19_ = 5.035, *p* = 0.018, one-way ANOVA; Tukey post hoc test *p* = 0.024; Figure 4A). For NMDAR subunits, a significant decrease in extra-synaptic GluN1 (F_2,26_ = 5.871, *p* = 0.008, one-way ANOVA; Tukey post hoc test *p* = 0.006; Figure 4D) and GluN2B (F_2,26_ = 5.179, *p* = 0.012, one-way ANOVA; Tukey post hoc test *p* = 0.010; Figure 4H) was observed from neonate age into adulthood.

**Figure 5:**
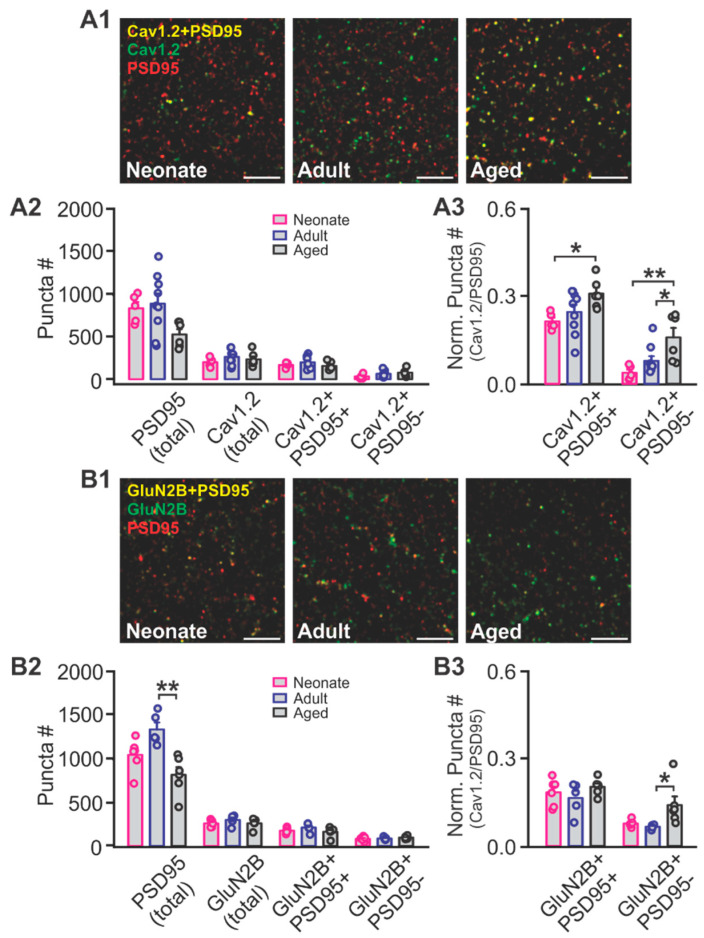
Age-dependent Cav1.2 and GluN2B expressions in the piriform cortex layer Ib. (**A1**) Example staining of Cav1.2 and PSD95 in neonate, adult and aged PC layer Ib. Scale bars, 5 µm. (**A2**) Puncta numbers of Cav1.2 and PSD95. (**A3**) Normalized punta numbers of Cav1.2 over PSD95. (**B1**) Example staining of GluN2B and PSD95 in neonate, adult and aged PC layer Ib. Scale bars, 5 µm. (**B2**) Puncta numbers of GluN2B and PSD95. (**B3**) Normalized punta numbers of GluN2B over PSD95. * *p* < 0.05; ** *p* < 0.01.

We then specifically looked at PC layer Ib LTCC and NMDAR expressions using immunohistochemistry and confocal imaging. We used co-labeling with post synaptic density protein 95 (PSD95) to index synaptic expression. The absolute numbers of Cav1.2 puncta (F_2,17_ = 1.013, *p* = 0.384), PSD95 co-localizing Cav1.2 (Cav1.2^+^/PSD95^+^, F_2,17_ = 1.184, *p* = 0.330) and non-PSD95 co-localizing Cav1.2 (Cav1.2^+^/PSD95^-^, F_2,17_ = 2.147, *p* = 0.147; One-way ANOVA; Figure 5A1,A2) were not different among age groups. However, there is a tendency of reduced PSD95 in the aged group which indicates a reduced number of synapses, consistent with previous reports [22,23]. When we normalized the Cav1.2 puncta to the PSD95 number, we observed increased relative Cav1.2 expression both synaptically (PSD95^+^; F_2,17_ = 3.764, *p* = 0.044) and extra-synaptically (PSD95^-^; F_2,17_ = 6.968, *p* = 0.006; Figure 5A3). Tukey post hoc tests showed that Cav1.2 was higher in aged animals compared to neonates both synaptically (*p* = 0.043) and extra-synaptically (*p* = 0.006). Aged animals also had higher Cav1.2 expression compared to adults at the extra-synaptic sites (*p* = 0.035).

Overall GluN2B patterns were not different among groups in layer Ib, while PSD95 was significantly reduced in the aged (F_2,14_ = 9.703, *p* = 0.002; Figure 5B1,B2). When normalized to PSD95 expression, extra-synaptic GluN2B (PSD95^-^) showed relative higher expression in the aged group (F_2,14_ = 4.619, *p* = 0.029; Figure 5B3), while there was no difference between neonate and adult animals (*p* = 0.936). The higher expression in extra-synaptic GluN2B in neonates by Western blotting likely reflects the heterogeneity of the tissue (different layers and cell types).

### 2.3. Ryanodine Mediated Ca^2+^ Signalling in Aged Rats

**Figure 6:**
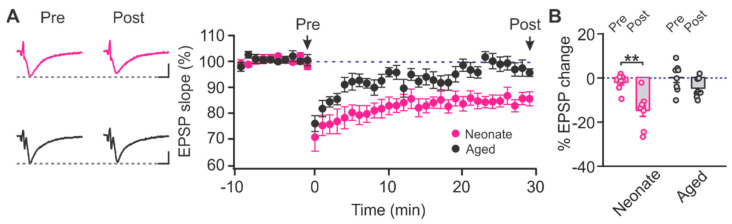
The role of ryanodine receptor in age-dependent LTD in the piriform layer Ib. (**A**) Example traces and time courses of fEPSPs with LTD inductions in the presence of dantrolene (50 µM) in neonate and aged rats. Scale bars, 0.5 mV, 5 msec. (**B**) % changes of fEPSPs at 30 min post-LTD induction. Neonate: *n* = 8; Aged: *n* = 8. ** *p* < 0.01.

Given the age-related increase in LTCC contribution to LTD induction within the PC, we explored downstream signalling pathways that could be involved in LTCC-mediated synaptic plasticity. Cav1.2-LTCCs can be located near junctions between the endoplasmic reticulum and the plasma membrane, where coupling through ryanodine receptors (RyRs) creates microdomains of Ca^2+^ signalling via Ca^2+^ induced Ca^2+^ release in both neuronal and non-neuronal cell populations [24]. To determine the specific role of RyRs in mediating LTCC-dependent LTD within the PC of aging rats, we induced LTD in the presence of RyR inhibitor dantrolene (50 µM) in both neonatal and aging animals. The pre-application of dantrolene inhibited LTD in aged rats (t = 2.007, *p* = 0.085, pre vs. post; paired *t*-test) but had no effect on the LTD observed 30 min post-induction in neonatal rats (t = 5.222, *p* = 0.001, paired *t*-test, pre vs. post; Figure 6). These data suggest that in aged rats, LTCCs may couple through RyRs to initiate downstream intracellular Ca^2+^ signalling essential to LTD.

### 2.4. The Contribution of GluN2B-Containining NMDARs to LTD in the Piriform Cortex

**Figure 7:**
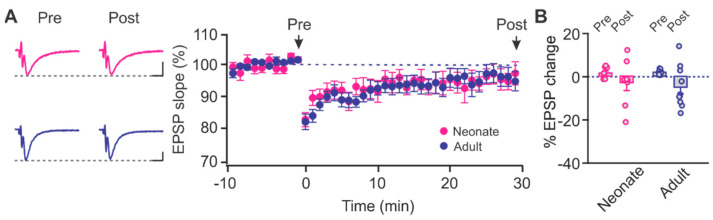
The role of GluN2B in age-dependent LTD in the piriform layer Ib. (**A**) Example traces and time courses of fEPSPs with LTD inductions in the presence of Ro25-6981 (1 µM) in neonate and adult rats. Scale bars, 0.5 mV, 5 msec. (**B**) % changes of fEPSPs at 30 min post-LTD induction. Neonate: *n* = 8; Adult: *n* = 9.

In various brain regions, there is an early developmental shift from GluN2B to GluN2A-containing NMDARs that coincides with synaptic maturation and is essential to the emergence of increased associative learning abilities [25,26]. A similar synaptic shift from GluN2B to GluN2A is also seen in PC neurons during olfactory-based learning and may contribute to network stability following odor experience [27]. Although both young and adult rats exhibited robust NMDAR-dependent LTD, we were curious to know whether there was a functional shift in the contribution of GluN2B-NMDARs in mediating LTD within the PC. We bath applied a specific GluN2B-NMDAR antagonist Ro 25-6981 (1 µM) prior to inducing LTD. Ro 25-6981 was significantly effective in blocking LTD in both neonates (t = 1.148, *p* = 0.289, pre vs. post; paired *t*-test) and adults (t = 2.255, *p* = 0.061, pre vs. post; paired *t*-test; Figure 7). Our data suggest there is significant contribution of GluN2B in mediating LTD in PC layer Ib from neonate into adulthood. However, we perceived no changes in the dependency on GluN2B-NMDARs in the PC.

## 3. Discussion

Here, we report an age-related change in the contribution of NMDARs and LTCCs to LTD of the PC. We find that slices from neonatal and adult rats exhibit a robust NMDAR-dependent LTD, that is unaffected by the LTCC antagonist nimodipine. Conversely, in slices from aged rats, we induced an NMDAR-independent LTD, which was inhibited by nimodipine, and reliant on ryanodine-mediated intracellular calcium release from intracellular stores. Higher relative Cav1.2 expression to PSD95^+^ synapses in the associational pathway of aged PC is consistent with the increased function of LTCCs in plasticity.

In the hippocampus, the threshold for inducing LTP increases, while susceptibility for LTD also increases with aging [18,20,21,28,29,30]. Both the decreased tendency for LTP and the increased tendency for LTD during aging are attributed to LTCC hyperfunction and concomitant reduced functionality of NMDARs [21]. Blocking LTCCs can reverse the susceptibility to LTD induction and enhance NMDAR-dependent LTP [31]. In adult rats, 1 Hz stimulation that elicits little LTD, produces robust LTD in the presence of an LTCC activator [32]. Whereas in the aged animals, the same low-frequency stimulation robustly evokes LTCC-dependent LTD [31,33]. However, in the PC, we did not observe different amount of LTD expression with the 1 Hz induction protocol at different ages. Nevertheless, similar to hippocampal LTD [19], PC layer Ib LTD undergoes a switch from NMDAR-dependency in the young to LTCC-dependency in the aged. There appears to be no inter-dependency of NMDARs and LTCCs involved in 1 Hz-LTD within the PC. The amount of LTD expressed following the application of nimodipine in neonatal and adult rats, or D-APV in aged rats, was similar to that induced in aCSF, suggesting no contribution of LTCCs or NMDARs to these age groups, respectively. Moreover, blocking NMDARs or LTCCs in young and aged rats, respectively, nearly completely prevented LTD. We hypothesize that this is an indication of diverse signalling pathways [20]. Whether this is true for other types of plasticity induced by other stimulation patterns remains to be tested.

Additionally, we show that RyRs mediate LTD in aged animals, likely through enhanced LTCC-RyR coupling in aging PC neurons. RyRs are gated by FK506 binding proteins (FKBP) [34]. FKBP stabilizes RyR by inhibiting RyR mediated Ca^2+^ release [35]. In the hippocampus, FKBP expression down-regulates and RyR expression up-regulates with aging [35]. The role of RyR-mediated Ca^2+^-induced Ca^2+^ release in LTCC-dependent LTD may be critical in understanding the paradoxical role of LTCCs in either promoting synaptic plasticity or contributing to calcium dysregulation [36]. Precise regulation of RyR-mediated Ca^2+^-induced Ca^2+^ signalling depends on a number of factors, including binding proteins such as FKBP [37], cationic channel coupling [24], as well as the regulation of LTCCs within supramolecular complexes at plasma membrane-endoplasmic reticulum junctions [38]. Though here we show that RyR-mediated calcium release is necessary for LTD, direct coupling between LTCCs and RyRs via FKBP in the context of synaptic plasticity of the PC has yet to be shown.

Alternative splicing of LTCCs may also be associated with aging [39], impacting activation and trafficking on the cell surface and modifying the LTCC-RyR interaction and the initiation of downstream calcium signalling. This rich diversity (of over 50 Cav1.2 splice combinations) may be highly variable in both developmental and aging cohorts, contributing to the overall manifestation of intracellular calcium signalling and release within neurons [40,41,42]. The impact of alternative splicing of LTCCs in the brain is still largely unexplored. The resulting modification of calcium balance, however, may not only drive synaptic plasticity or dysregulation, but may also be attributed to changing thresholds required for LTP or LTD induction with age.

The functional impact of this increase in LTCC-mediated synaptic plasticity is still unclear. LTCCs have been posed as a major source of calcium entry leading to dysregulation of calcium homeostasis during aging, which can lead to cognitive decline and spatial memory dysfunction [21,33]. On the other hand, assessments of spatial learning behaviours in aged rats suggests that high cognitive function can be maintained in aged rats by voltage-gated calcium channel (VGCC)-dependent LTP [18] and LTD [19], arguing that this form of plasticity is compensatory and neuroprotective. However, these studies are limited to assessments of certain aspects of learning and may not capture the full impact of memory formation under LTCC-mediated synaptic plasticity. Besides learning acquisition, memory duration and input-specificity can be impacted by LTCC functioning. In an early odor conditioning involving the PC, for example, the activation of LTCCs following a block of NMDAR-mediated synaptic plasticity restored both short-term and long-term preference memory, but did not rescue impaired discrimination between conditioned and similar odor mixtures in the absence of NMDARs [6]. Further examination of LTCC-mediated synaptic plasticity within different learning paradigms is necessary to fully grasp functional consequences for different types of spatial and associative memory formation during aging.

The particular roles of PC LTD in olfaction and olfactory learning remain to be tested. In other model systems, LTD is necessary for behavioral flexibility [43,44]. Transgenic mice with phosphatase 2A inhibition [44] or synaptotagmin-3 knock out [43] have specific impairment in Schaffer collateral synaptic LTD but exhibit intact LTP induction in the same synapses. These mice showed normal learning in locating a hidden platform in a Morris water maze but were slower in learning when the platform was re-located to the opposite quadrant of the maze [44]. Learning new information during a task may be impaired if LTD is not intact. De-potentiation of the previous learning-encoding synapses is necessary for the learning of new information [43]. The importance of LTD has also been demonstrated in spatial memory consolidation [45], and a visual object discrimination task that is dependent on perirhinal cortex [46], consistent with the theory that decreases in synaptic efficacy may either prevent synaptic saturation through synaptic scaling [47], or increase the signal to noise ratio by depressing non-potentiated synapses or priming these synapses for LTD [44,47,48]. LTD in the PC may be associated with some of these processes as well. The role of synaptic depression in the PC has been previously described in non-associative forms of learning, such as odor adaptation [49,50], though these are mediated by metabotropic glutamatergic receptors. The roles of LTCC-dependent LTD in both PC and other learning encoding structures warrant further investigation.

## 4. Materials and Methods

### 4.1. Subjects

Neonatal (1–2 weeks), adult (2–3 months) and aged (20–25 months) Sprague Dawley rats of mixed sexes were used. Rats were kept in a standard 12 h light-dark cycle, with food and water ad libitum. Experimental procedures were approved by the Institutional Animal Care Committee at Memorial University of Newfoundland and followed Canadian Council’s guidelines on Animal Care.

### 4.2. In Vitro Slice Preparation

Parasagittal slices were obtained from Sprague Dawley rats from 3 different age cohorts. Rats were anesthetized with isofluorane and decapitated for brain extraction. Brains were sliced using a vibratome (Leica VT-1200s) in ice cold HEPES artificial cerebrospinal fluid (aCSF; in mM, 92 NaCl, 2.5 KCl, 1.2 NaH_2_PO_4_, 30 NaHCO_3_, 20 HEPES, 25 glucose, 5 sodium ascorbate, 2 thiourea, 3 sodium pyruvate, 10 MgSO_4_, 0.5 CaCl_2_) bubbled with carbogen gas (95% O_2_/5% CO_2_). For improved tissue health, slices were recovered in N-methyl-D-Glucamine (NMDG) based recovery solution (in mM, 93 NMDG, 2.5 KCl, 1.2 NaH_2_PO_4_, 30 NaHCO_3_, 20 HEPES, 25 glucose, 5 sodium ascorbate, 2 thiourea, 3 sodium pyruvate, 10 MgSO_4_, 0.5 CaCl_2_) for 10–12 min at 34 °C before moving to back to HEPES recovery solution. Following 30 min, slices were transferred to recording aCSF (in mM, 124 NaCl, 2.5 KCl, 1.2 NaH_2_PO_4_, 24 NaHCO_3_, 5 HEPES, 12.5 glucose, 2 MgSO_4_, 2 CaCl_2_) at room temperature.

### 4.3. Electrophysiological Field Recordings

Slices were continuously perfused with bubbled aCSF in an open bath recording chamber and visualized with an Olympus BX51WI microscope. Temperature of the recording bath was maintained at 28–30 °C. To measure fEPSPs, a concentric stimulation electrode was placed in layer Ib of the piriform cortex with a recording electrode (1–2 MΩ) filled with aCSF placed within 500 µm. Test stimulations of 0.2 ms were given every 20 s and slices were given 20–30 min to equilibrate. Stimulation intensity was adjusted from 0–200 µA to obtain an input-output relationship, and baseline stimulations were chosen at 70–80% of the maximum fEPSP slope. PPRs were obtained by recording 10 paired stimulations with a 50 ms interval. After a stable baseline for 10–20 min, we induced LTD by stimulating at 1 Hz for 15 min (900 stimulations) and recorded 30 min post LTD induction. Drug application was applied after a stable baseline was achieved and was continuously washed in for 10 min (D-APV (50 µM), Nimodipine (10 µM)) or 20 min (Ro 25-6981 (1 µM), Dantrolene (50 µM)) as well as during LTD induction. Dantrolene was continuously washed in post LTD induction. D-APV, Ro 25-6981, and Dantrolene were obtained from Tocris Biosciences, while Nimodpine was obtained from Sigma-Aldrich. Data were acquired using Multiclamp 700B (Molecular devices), filtered at 2 kHz and digitized at 10 kHz using pClamp 10.5 software (Molecular Devices). Field EPSP peak amplitude and slope were determined offline in ClampFit 10.5 (Molecular Devices).

### 4.4. Synaptic and Extra-Synaptic Extraction

Following decapitation, PC tissue was extracted, flash-frozen on dry ice, and stored at −80 °C for processing. Samples were homogenized in 300µL sucrose buffer (320 mM sucrose, 10 mM Tris buffer (pH 7.4), 1 mM EDTA, 1 mM EGTA, 1× complete protease inhibitor cocktail and phosphatase inhibitor cocktail (Roche)), and then centrifuged at 2000 rpm (224× *g*) at 4 °C, for 15 min. The supernatant was collected, containing cytosolic, synaptic and extra-synaptic fractions, and was centrifuged at 10,000 rpm (5600× *g*) at 4 °C, for 30 min to obtain a pellet. For detergent extraction, 200 µL of Triton X-100 buffer (10 mM Triton-100, 10 mM Tris buffer (pH 7.4), 1 mM EDTA, 1 mM EGTA, 1× complete protease inhibitor cocktail and phosphatase inhibitor cocktail (Roche)) was added to the pellet, and then vortexed and incubated at 4 °C for 30 min with gentle rotation. The suspension was then centrifuged at 14,000 rpm (10,976× *g*) at 4 °C, for 2 h. The resulting pellet consisted of postsynaptic densities and synaptic junctions that is insoluble in Triton X-100 [51]. The pellet was resuspended in 50 μL of sodium chloride-Tris-EDTA (STE) buffer (100 mM Tris buffer (pH7.4), 10 mM EDTA, 10% SDS, 1× complete protease inhibitor cocktail and phosphatase inhibitor cocktail (Roche)) and labelled as the synaptic fraction. The mixture was sonicated and stored at −80 °C. The supernatant containing the extra-synaptic fraction was diluted with acetone (6 volumes), and then stored at 4 °C overnight. The following day, the suspension was centrifuged at 5000 rpm (1400× *g*) at 4 °C, for 15 min. This pellet, the extra-synaptic fraction, was resuspended with 50 μL of STE buffer, sonicated and stored at −80 °C.

### 4.5. Western Blotting

Standard BCA assay (Pierce) was used to quantify total protein concentration. The amount of lysate was calculated to make 40 μg (extra-synaptic) and 10 μg (synaptic fractions) of protein for each sample. Lysate solution, sample buffer (0.3 M Tris-HCl, 10% SDS, 50% glycerol, 0.25% bromophenol blue, 0.5 M dithiothreitol), and dH_2_O were prepared and boiled (2 min, 100 °C).

Samples and pre-stained protein ladder (PM008-0500, Froggabio) were loaded into a 7% SDS-PAGE gel. Following separation, the samples were transferred to nitrocellulose membranes (0.45 μm; Thermo Fisher Scientific), which were stained with reversible Ponceau S staining. The membranes were rinsed in 1× Tris Buffered Saline with 0.1% Tween 20 (TBST) for 10–15 min and were blocked for 2 h with 5% non-fat dry milk at room temperature prior to incubating at 4 °C overnight with the following antibodies: rabbit Anti-GluN2A (1:5000; cat. no. 07-632; Millipore Sigma), rabbit Anti-GluN2B (1:5000; cat. no. 06-600; Millipore Sigma), rabbit Anti- GluN1 (1:5000; cat. no. D65B7; Cell Signalling) and rabbit Anti-Cav1.2 (1:2000) [52]. The following day, membranes were washed (3 × 5 min with 1× TBST) and applied with Anti-rabbit horseradish peroxidase-bounded second antibody (1:10,000; Thermo Scientific, Bratislava, Slovakia) in 1× TBST at room temperature for 1.5 h, and washed (3 × 10 min 1× TBST).

Using chemiluminescent substrate, protein bands were visualized, and optical density (OD) measured using ImageJ software. Density was normalized to the total protein density observed by Ponceau staining.

### 4.6. Immunohistochemistry

Rats were perfused transcardially with 4% paraformaldehyde (PFA) in a 0.1 M phosphate buffer. Brains were extracted and stored in 4% PFA for 24 h and then transferred to a 0.1 M phosphate-buffered saline (PBS) at 4 °C. Coronal sections (50 µm) were cut (Leica VT 1000S or Compresstome VF-310-OZ), and transferred to 24 well plates containing PVP solution, and were stored at 4 °C until further processing.

Slices were washed 3 × 5 min in Tris buffer (0.1 M, pH 7.6) and blocked in 1 mL of 5% bovine serum albumin (BSA) in Tris buffer for 1 hr at room temperature. A primary antibody (FP1 rabbit Anti-Cav1.2 antibody (1:500) [52], or Anti-GluN2B (1:2000; cat. no. AGC-003; Alomone Labs) was applied in 5% BSA in Tris buffer on shaker at 4 °C overnight.

The following day, the sections were washed 2 × 10 min in Tris buffer. All subsequent processes took place in the dark. Sections were probed with Anti-rabbit secondary antibody Alexa 647 (1:500; cat. no.A-21244; Thermo Fisher Scientific) in 5% BSA in Tris buffer, for 1 h, at room temperature on low speed shaker. Afterwards sections were washed 3 × 5 min in Tris buffer followed by 1 × 10 min wash in Tris A (0.1% Triton-X-100 in Tris buffer) and 1 × 10 min wash in Tris B (0.1% Triton-X-100 and 0.005% BSA in Tris buffer). Thereafter the sections were incubated with the second primary antibody; mouse Anti-PSD95 antibody (1:2000; cat. no. MA1-046; Invitrogen) in 5% BSA in Tris B on shaker at 4 °C overnight.

On the last day, sections were washed 1 × 10 min in Tris A followed by 1 × 10 min in Tris B. They were probed with Anti-mouse secondary antibody Alexa 555 (1:500; cat. no. A-21424; Thermo Fisher Scientific) in 5% BSA in Tris B, for 1 h, at room temperature on low-speed shaker. Sections were then washed 3 × 5 min in Tris buffer and mounted on chrome-gelatin coated slides. Nuclei were counterstained with 4′-6-diamidino-2-phenylindole (DAPI; cat. no. ab104139; abcam) and slides were cover slipped. They were kept at 4 °C before confocal microscopy scanning.

### 4.7. Confocal Imaging and Analysis

Using an FV10i confocal microscope (Olympus), z-stacks (∼0.4 mm^2^) were taken from the dendritic layer Ib of the PC at a magnification of 60× with a 10× zoom. Z-stacks were acquired from three to four sections from rostral to caudal area. Photomultiplier tube assignments, confocal aperture size, and contrast remained constant for all sections.

Otsu method in image thresholding was used to perform automatic image segmentation into foreground and background. Using Grana’s (BBDT) algorithm, connected components were identified. In order to remove noise of images, two filters of size 11 × 11 and 5 × 5 were applied resulting in creating two masks. Using the logical ‘AND’ operator between these two masks identified regions of overlap between two channels. Next, by using Grana’s (BBDT) again, the overlapped regions of two images were counted.

### 4.8. Statistics

All statistics were conducted in OriginPro 9.0 (OriginLab Corp). For >2 group comparisons, one-way or two-way ANOVA were used, followed by post hoc Tukey. Paired *t*-tests were used to measure the effects of LTD inductions. Differences between groups were considered significant when *p*-values were <0.05.

## Data Availability

Data are contained within this article or Supplementary Material.

## Supplementary Materials

The following are available online at https://www.mdpi.com/article/10.3390/ijms222413551/s1.
