# Supplementary figures and images for: Age-Dependent Contributions of NMDA Receptors and L-Type Calcium Channels to Long-Term Depression in the Piriform Cortex
Source: Int J Mol Sci. 2021 Dec 17;22(24):13551. doi: 10.3390/ijms222413551 (PMC8706958; doi:10.3390/ijms222413551)

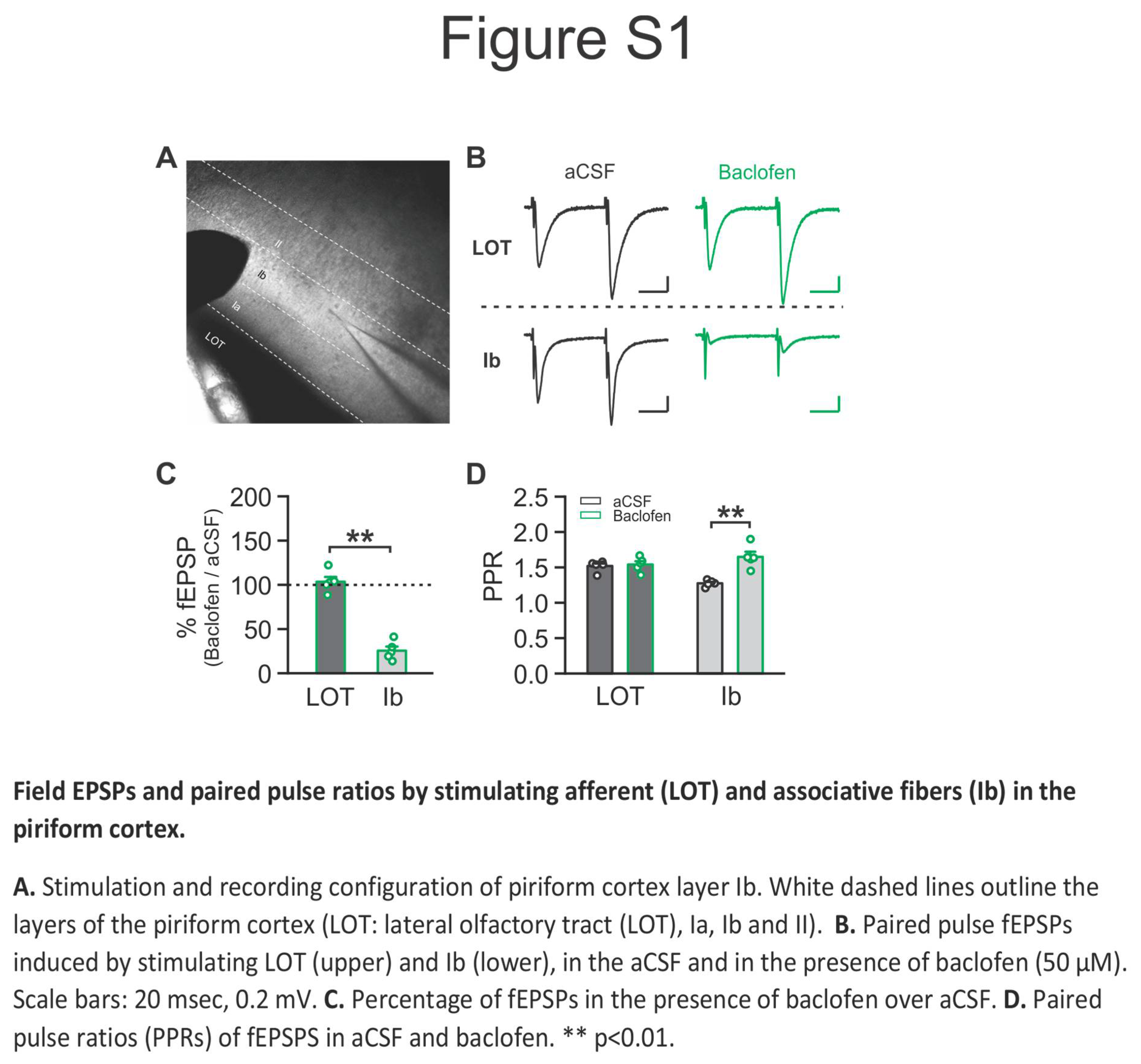

Supplement: Supplementary file 1 [file ijms-22-13551-s001.zip › ijms-1501997-supplementary.png]
